# Supplementary material for: Patterns of Intron Gain and Loss in Fungi
Source: PLoS Biol. 2004 Nov 30;2(12):e422. doi: 10.1371/journal.pbio.0020422 (PMC532390; doi:10.1371/journal.pbio.0020422)
Supplement: Table S1 — Also available at http://genes.mit.edu/NielsenEtAl/. (4.3 MB ZIP). [file pbio.0020422.st001.zip › NielsenEtAl/html/581.html]

AN7936.1.NCU07938.1.MG04153.1.FG09737.1


```
 CLUSTAL W (1.82) Multiple Sequence Alignments - Introns Inserted


Sequence 1: NCU07938.1	637 aa
Sequence 2: MG04153.1	552 aa
Sequence 3: FG09737.1	516 aa
Sequence 4: AN7936.1	522 aa
Alignment Length: 642 aa
Number Identitical Residues: 147 aa
Alignment Score (without introns) 9128


MG04153.1 	MANN--GEAPPESTSQTSLDDSAKALQA------------------SPVGGPGVWSTSGT
NCU07938.1	MSGSRPSSLTSEASVGRAVDSSNFPVQVQQPTRAPAQEKEDVISTISPSNDPTTYQPGPV
FG09737.1 	MAEKDENRPSPDYSGSHRHDIDAISVQS------------------SLSDAPTARDTTSE
AN7936.1  	------------------------------------------------MASPQTPSK---
          	                                                   * . .    

MG04153.1 	STPKTD-DP--SQSDDGPPTDLEG-LHRESTGPVYSTFSTLNKKWIIGMVSFSSFISPMT
NCU07938.1	PVPNPDPDPNLDTSDINASQDPEANLTRLSSGPAYTVFPPSTRKWIVTMITFAGFISPMT
FG09737.1 	KLGESMNNGISAQVDEGILGDPEN-MTRVSSGPAYSTFSNNTKRWILALVTAASFVSPMT
AN7936.1  	------------EGDTGLKGGLDVVATNTTGEVPYSVFTKAQKRYIVFFASWAGFFSPVS
          	              * .   . :    . :    *:.*.   :::*: : : :.*.**::

MG04153.1 	ANIYFPSITPIANELGVSIGLMNLTITTYM~IMQGLSPTIIGDLGDMTGRRPAFIIAFTI
NCU07938.1	ANIYFPVLDAVARDLNVSISKANLTLTTYM~IMQAFSPTLYGDFGDAAGRRPAFIISFIV
FG09737.1 	ANIYFPALNPIADDLNISISLINLTLTSYM~IFQGLSPTIFGDFGDMAGRRPAYIVAFSI
AN7936.1  	SQIYFPALNSIADDLGVTSALINLTLTSYM0IFQGVSPMFVGDFADKAGRRPAYMVCFLI
          	::**** : .:* :*.:: .  ***:*:** *:*..** : **:.* :*****:::.* :

MG04153.1 	YIVANIGLALQSNYAALLVLRCVQGAGSSGTLALGFAVVADLAVSAERGKYMGFVGAGIQ
NCU07938.1	YICANLGLALQKNYAALLVLRMLQSFGSSGSLALCYAVVADVAVSAERGKYMGFVSAGIN
FG09737.1 	YIGANIGLALQRNFVALLILRCVQSAGSSGTLALGYAVVADISSTGERGKYMGIVGAGIN
AN7936.1  	YIAANIGLALQDNFAALFVLRCLQSAGSSTTIALSAGVVSDIAIAAERGSYMGFVTAGSL
          	** **:***** *:.**::** :*. *** ::**  .**:*:: :.***.***:* **  

MG04153.1 	VGPSIGPVIGGALSEYLGWRSIFWFCAILTIVWLIPFILAVPETCRKVVGNGSVPPQG-W
NCU07938.1	IGPSLSPVIGGVIGQYLGWHAIFWFCLIYTGVWLVPYVLLVPETCRNVVGNGSTKPQAWW
FG09737.1 	IGPALGPLLGGILSQYLGWPAIFWFCAIFSAAWMIPFILSAPETCRNVVGNGSIAPQK-W
AN7936.1  	LGPAMGPVIGGLLSQYLGWRSVFWFLTIFAGAFLVPFVLLFPETARAIVGNGSIPPPK-W
          	:**::.*::** :.:**** ::***  * : .:::*::*  ***.* :*****  *   *

MG04153.1 	NRTLVDYIQ-------ERRHPTD-------------RPETPKRKFYFPNPFKTLAVVFEK
NCU07938.1	NMTVVDYVKGRKRRRRRRRHPPGSREEEEEEEEAKRQAERTRPKLRFPNPFNTLKVVFNP
FG09737.1 	NRTLLDIYS-------HRGDVPD--------------QNVPKRKLKFPNPLKTLYIVFDK
AN7936.1  	NIPLLTLFQ-------ARKEPQP--------------EDFDWPKLRFPNPIRTLSIVFQK
          	* .::   .        * .                  :    *: ****:.** :**: 

MG04153.1 	DLGLLLMLNASVYLMFMLILATMSPQLHSIYNLNEFQVGLCYLPYGFGCFFSAIVQGYLL
NCU07938.1	DLGLLISYGTMVYLVFILICATLGTQFADRYGYNQLETGLCYLPYGIGCTVAAVVQGRIL
FG09737.1 	EMAIILCINAIIYVAFILTAATLSTLFKEIYGYNDLQVGLCYLPYGFGCAFAVVAQGRVL
AN7936.1  	DIAIILIANAILFAGFYDVTAAIPSIYNELYGLDDLYIGLCYVPFGLGATVASIATGKLL
          	::.:::  .: ::  *    *:: .   . *. :::  ****:*:*:*. .: :. * :*

MG04153.1 	DWNYRRTARKVGMKIDMKRGDDLTKFPIEKARIQLIAPMLGIGIISTICYSWIMQTEQAL
NCU07938.1	DWNYRRWAKKMGMTIDYKRGDDLSKFPIERARLQPVIPILGIGVVATIGYAWALETTTHV
FG09737.1 	DWNYRRIAKAIGFTIDRKRGNDLAKFPIETARIQAIYPILLAGIVTLIGYGWALQVETSV
AN7936.1  	DFNYRRLAKQLNVPLQETRARNLTHFPIEFARLQVALPLLTLGAFTIIAFGWCLNYGVHL
          	*:**** *: :.. :: .*. :*::**** **:*   *:*  * .: * :.* ::    :

MG04153.1 	GLVLFFGFLIGVTVNGSFSMMNTLIVDLYTDAPATAVAANNL~-----FRCSLGAICMSF
NCU07938.1	AGPLVLLFVVGLCVTGSFSLVNTLVVDLYPEAPATAVAATNL~-----VRCLCGAGATAF
FG09737.1 	AVPLVLVFLIGMFVPTSFSVLNTLIVDLNPNAPATATAANNL~-----VRCMFGAASTAA
AN7936.1  	AAPTTILFLMGLTLTGAFNTVSTLLVDFYPSNASAATASNNL0DDVSRLESQYRLCLATT
          	.    : *::*: :  :*. :.**:**: .. .::*.*:.** .. : ...       : 

MG04153.1 	IENMVQTMGRGWCYTF~WALLTASLM~PILWLLVQRGP~RWRE----------ERRLRKE
NCU07938.1	IEAMLQKMGVGWTFTF~WPLVLVVFS~PIIYLLVTRGP~GWRAQRKARFQRERERREQKE
FG09737.1 	IDHMIEGMGR------~---------~----------E1VWDG----------EPQKPSE
AN7936.1  	IDNCSHWMLPSHVVGK0STAMGVGTR2TLSPSTFTALC~ANHG----------LQLENSD
          	*:   . *  .       .        .                              .:

MG04153.1 	KIAQKELAQQDAEKSGK------------------------------
NCU07938.1	RSANEESLESSDNSSASVEKTEEGDGEKRMVDGIFVTEAKKGQGLSS
FG09737.1 	KH---------------------------------------------
AN7936.1  	EPAYMHSIEITPLLGCLELAFE-------------------------
          	. :     .     .      .
```
